# Relation between intra-abdominal pressure and early intestinal ischemia in rats

**DOI:** 10.1136/tsaco-2020-000595

**Published:** 2020-12-01

**Authors:** Steven G Strang, Ben van der Hoven, Kim Monkhorst, Samir Ali, Esther M M van Lieshout, Oscar J F van Waes, Michael H J Verhofstad

**Affiliations:** 1 Trauma Research Unit, Department of Surgery, Erasmus MC, University Medical Center Rotterdam, Rotterdam, The Netherlands; 2 Department of Intensive Care Medicine, Erasmus MC, University Medical Center Rotterdam, Rotterdam, The Netherlands; 3 Department of Pathology, Josephine Nefkens Institute, Erasmus MC, University Medical Center Rotterdam, Rotterdam, The Netherlands

**Keywords:** multiple trauma, animal experimentation, abdominal injuries, compartment syndromes

## Abstract

**Background:**

Little is known on early irreversible effects of increased intra-abdominal pressure (IAP). Therefore, timing of abdominal decompression among patients with abdominal compartment syndrome remains challenging. The study objective was to determine the relation between IAP and respiratory parameters, hemodynamic parameters, and early intestinal ischemia.

**Methods:**

Twenty-five anesthetized and ventilated male Sprague-Dawley rats were randomly assigned to five groups exposed to IAPs of 0, 5, 10, 15, or 20 mm Hg for 3 hours. Respiratory parameters, hemodynamic parameters, and serum albumin-cobalt binding (ACB) capacity as measure for systemic ischemia were determined. Intestines were processed for histopathology.

**Results:**

IAP was negatively associated with mean arterial pressure at 90 (Spearman correlation coefficient; Rs=−0.446, p=0.025) and 180 min (Rs=−0.466, p=0.019), oxygen saturation at 90 min (Rs=−0.673, p<0.001) and 180 min (Rs=−0.882, p<0.001), and pH value at 90 (Rs=−0.819, p<0.001) and 180 min (Rs=−0.934, p<0.001). There were no associations between IAP and lactate level or ACB capacity. No histological signs for intestinal ischemia were found.

**Discussion:**

Although increasing IAP was associated with respiratory and hemodynamic difficulties, no signs for intestinal ischemia were found.

**Level of evidence:**

Prognostic and epidemiologic study, level II.

## Introduction

Intra-abdominal pressure (IAP) is the pressure concealed within the abdominal cavity. A substantial increase in IAP ≥12 mm Hg is termed intra-abdominal hypertension (IAH). If IAH exceeds 20 mm Hg, reduced intra-abdominal arterial perfusion pressure will result in organ dysfunction which is termed abdominal compartment syndrome (ACS). This condition is related to high morbidity and mortality.^1 2^ Risk factors and treatment options for ACS have been listed by the World Society of the Abdominal Compartment Syndrome in the “consensus definitions and clinical practice guidelines”.^3^ According to these guidelines, a decompression laparotomy should be performed if non-invasive measures failed.

An IAP of 20 to 25 mm Hg for a period of only 60 min may already reduce mucosal blood flow of the intestines of rats and deteriorate intestinal-barrier function.^4^ Moreover, IAH may cause irreversible intestinal ischemia before alterations in cardiac output or mean arterial pressure (MAP) become noticeable.^5 6^ Therefore, *early* surgical decompression before the development of ACS is becoming increasingly common.^7^ Since this treatment is related to high morbidity, the surgeon must be sure whether organ dysfunction is caused by IAH.^8^

The detrimental effects of ACS and persistent high IAP are well known.^9^ However, the early irreversible effects of subclinical or transient IAH with intra-abdominal pressures up to 20 mm Hg are unknown. Knowledge of such effects may help surgeons in decision-making on early surgical abdominal decompression. As it is hardly feasible to study this in humans, an animal model should be used. The aim of this study was to determine the relation between early increased IAP and respiratory parameters, hemodynamic parameters, and the development of intestinal ischemia in rats.

## Methods

All animal experiments were performed in accordance with the recommendations of the Guide for the Care and Use of Laboratory Animals, and under the regulation and permission of the local Animal Care Committee. Adult (8–10 weeks old) male Sprague-Dawley rats (300–350 g, specific pathogen-free; Harlan Laboratories, Boxmeer, The Netherlands) were supplied with standard laboratory rat chow and water ad libitum, housed per two/three in individually ventilated cages, maintained on a 12:12 hours light–dark cycle, and acclimated for at least 1 week before the experiment. This manuscript was reported in line with the ARRIVE statement.^10^

### Experimental model and IAH induction

Twenty-five anesthetized and ventilated rats were randomly assigned to five groups and exposed in random order to an IAP of 0, 5, 10, 15, or 20 mm Hg for 3 hours. From a pilot study, it was known that exposing rats to higher levels of IAP or for a prolonged period of time was not feasible in this model. This was due to the detrimental effects on hemodynamic and respiratory parameters. Rats were anesthetized with intra-peritoneal ketamine hydrochloride (50 µg/g), ventilated following tracheostomy, and kept warm by a warming pad and tin foil. A capnograph and pulse oxymeter (both Siemens SC9000 XL monitor; Siemens Medical Systems, EM-PCS, Danvers, USA) were installed to measure end-tidal CO_2_ concentrations in expired air (EtCO_2_) and oxygen saturation. The carotid artery and internal jugular vein were cannulated (PE-50) for blood draw access and monitoring the arterial blood pressures (systolic, diastolic, and mean), heart rate (HR), and central venous pressure (CVP). The tail vein was cannulated for anesthetic and fluid infusion (10 µL per gram body weight of KMA mix, consisting of 0.72 mL 100 mg/mL ketamine, 0.08 mL 1 mg/mL medetomidine, and 0.3 mL 0.5 mg/mL atropine, in 20 mL normal saline). Administration of antibiotics was considered as non-contributing since the experiment lasted only 3 hours. An intraperitoneal catheter (12 Ch Redon drain) was placed for fluid instillation and IAP monitoring by a midline laparotomy. The abdomen was closed with a running suture, including all layers of the abdominal wall.

The model used in this study has been described in detail previously.^11^ All animals were allowed to stabilize for 30 min before baseline measurements of MAP, CVP, HR, end tidal CO_2_ (EtCO_2_), and saturation. Direct continuous measurement of IAP was performed via the intraperitoneal catheter. After baseline analysis, the IAP was increased by instillation of warmed (40°C) Gelafundin (gelafundingelatine polysuccinate 4%; B. Braun Medical B.V., Oss, The Netherlands) and placing the fluid bag on specific level. A plaster cuff was applied to the abdomen of rats in order to reduce the required volume of Gelafundin (not in original model). The IAP and positive end expiratory pressure were kept at the same level during the experiment. Ventilatory and hemodynamic adaptations were made to compensate for deterioration during IAH. The respiratory rate and peak inspiratory pressure were increased to maintain EtCO_2_ between 4.5 and 6.0 kPa. MAP was kept between 70 and 110 mm Hg by Voluven administering (6% hydroxyethyl starch 130/0.4 in 0.9% sodium chloride; Fresenius Kabi B.V. Zeist, The Netherlands). After completion of the experiment, all animals were sacrificed by exsanguination.

### Blood sampling

Blood samples (with a capillary) were drawn at baseline, at 90 and 180 min for analyzing blood gases and serum lactate (ABL800 FLEX analyzer; Radiometer, Copenhagen, Denmark). All analyses were done once. At the same time points, single blood samples (0.6 mL) were drawn for duplicate determination of albumin-cobalt binding (ACB) capacity according to Bar-Or *et al*, as a measure of systemic ischemia.^12^ This biomarker is a highly sensitive and rapid marker for ischemia, but it is non-specific for tissue type and therefore a marker for systemic ischemia. The ACB test is a low-cost test which is easy to perform and well available. Also, the assay showed promising results for the detection of ischemia in an animal model of ACS.^13^ The ACB test indicates systemic ischemia when its absorbance reaches above 0.4 absorbance units (ABSU), measured using a microplate reader (Wallac 1420 Victor2; Perkin Elmer, Groningen, the Netherlands).

### Histological examination

For each rat, five cross-sectional samples were taken at random locations of the intestine. If macroscopic damage was visible, samples were taken there as well. Samples were fixed with 10% formalin, embedded in paraffin and sliced in 4–5 µm sections, stained with routine H&E, and examined under a light microscope by a pathologist (KM) and clinical researcher (SGS); discrepancy was discussed. Histopathology was graded according to the Parks and Chiu/Park scoring systems for intestinal mucosal injury (Parks score for inflammation and necrosis and Park/Chiu score for mucosal lifting).^14–16^ All samples were scored at the most extensively affected areas. Mean scores of the five samples were calculated.

### Statistical methods

The sample size calculation was based on an expected correlation coefficient between IAP and Parks/Chiu score of 0.95. With an alpha error of 0.05 (two-tailed) and a beta of 0.2, five animals per group were needed. Data for all animals were analyzed using SPSS statistical software, V.21.0. All data were non-parametric and are displayed as median with corresponding quartiles (P_25_–P_75_). Kruskal-Wallis test was used in order to test differences in body weight between groups. Spearman rank correlation tests was used in order to test the association between IAP and the individual variables.

## Results

### Basic characteristics

**Figure 1:**
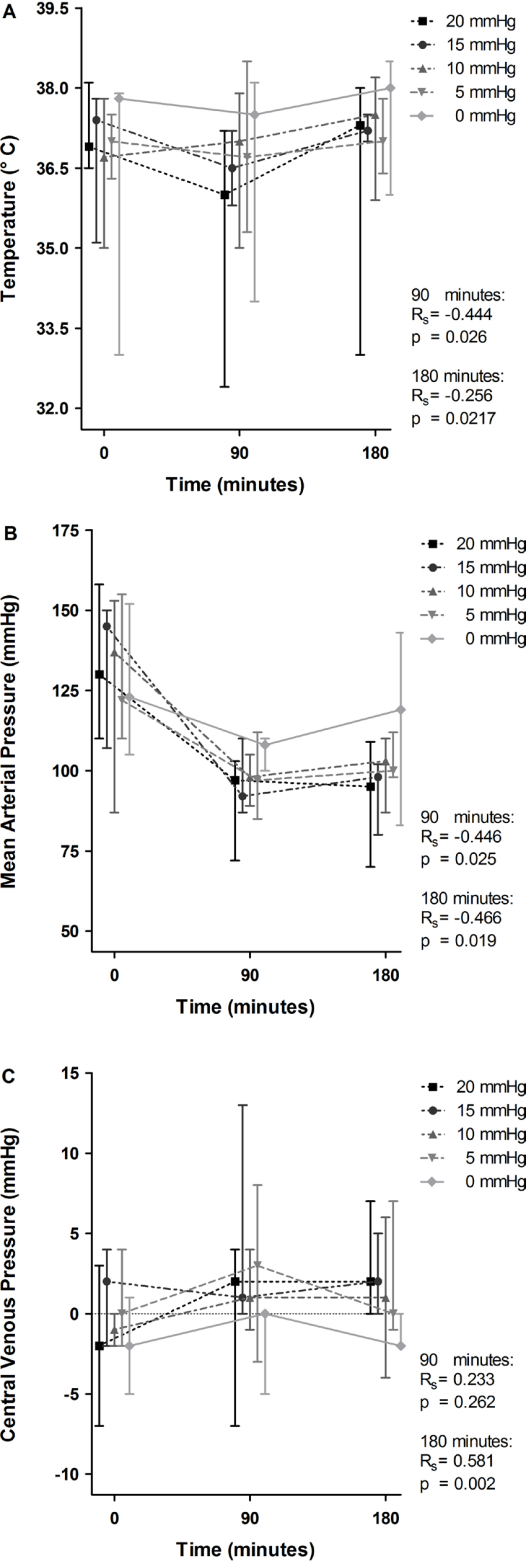
Effect of intra-arterial pressure (IAP) increase on temperature, mean arterial pressure and central venous pressure at 90 and 180 min. Temperature (A), mean arterial pressure (B) and central venous pressure (C) at 0, 90, and 180 min are demonstrated for the individual IAP groups and displayed as median with upper and lower limits. The Spearman correlation coefficient (Rs) and p value (p) represent correlation between IAP and the individual variables with corresponding statistical significance at 90 and 180 min.

The median body weight of the rats was 377 g (P_25_–P_75_, 368–392 g), and there was no association between body weight and the IAP group the rats were assigned to (p=0.767). The median body temperature decreased statistically significantly to 36.0°C at 90 min (figure 1A). At that time point, temperature was negatively correlated with IAP (Spearman rank correlation coefficient; Rs=−0.444, p=0.026). At 180 min, body temperature had normalized again. A decrease in the median MAP was noted in all groups (figure 1B). MAP was negatively correlated with IAP, both at 90 min (from MAP 108 mm Hg in the control group to 97 mm Hg in the highest IAP group; Rs=−0.446, p=0.025) and at 180 min (from MAP 119 mm Hg to 95 mm Hg, respectively; Rs=−0.466, p=0.019). CVP remained fairly constant across time in all groups (figure 1C). At 180 min, the median CVP ranged from −2 mm Hg in the control group to 2 mm Hg in the highest IAP group (positive correlation with IAP, Rs=0.581, p=0.002). All animals completed the experiment and were used in the analyses.

### Respiratory characteristics

**Figure 2:**
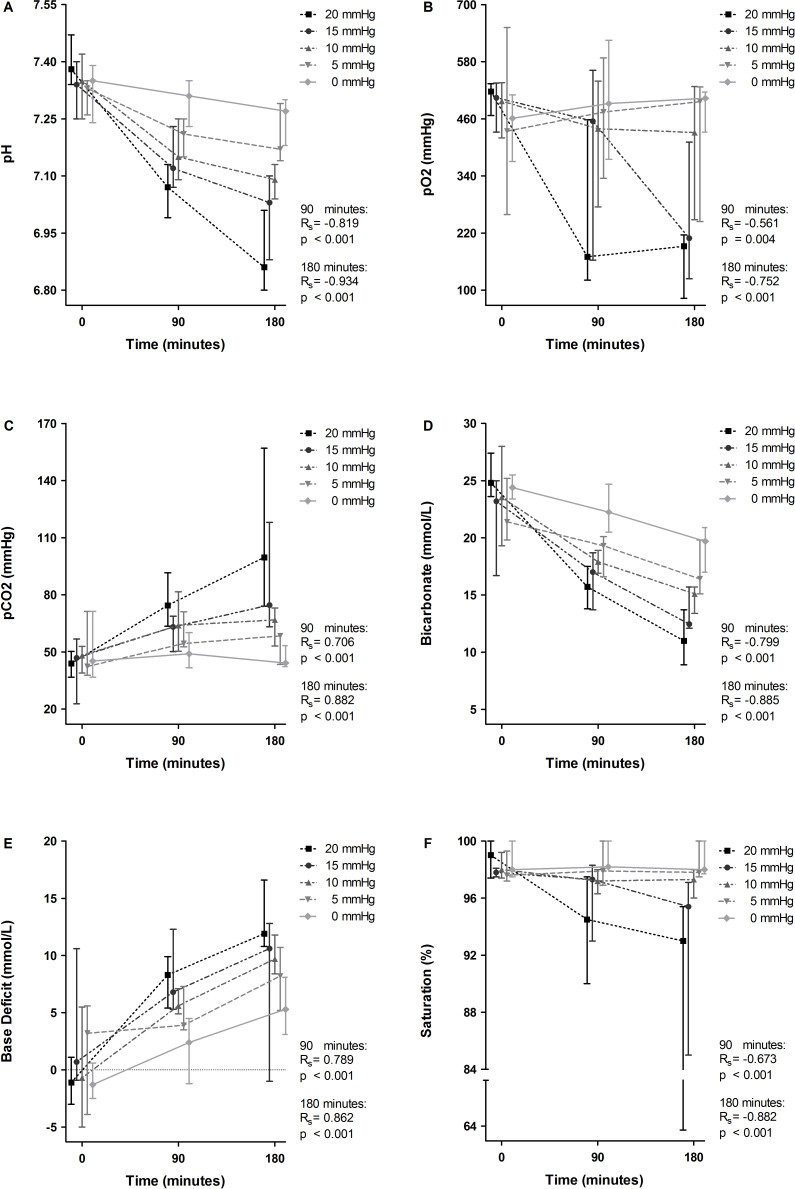
Effect of intra-arterial pressure (IAP) increase on arterial blood gas values at 90 and 180 min. pH (A), pO_2_ (B), pCO_2_ (C), bicarbonate (D), base deficit (E), and saturation (F) at 0, 90, and 180 min are demonstrated for the individual IAP groups and displayed as median with upper and lower limits. The Spearman correlation coefficient (Rs) and p value (p) represent correlation between IAP and the individual variables with corresponding statistical significance at 90 and 180 min.

IAP was positively correlated with median EtCO_2_ at 180 min (from 4.5 kPa in the control group to 5.4 kPa in the highest IAP group; Rs=0.639, p=0.001). Respiratory deterioration was reflected in all individual parameters of arterial blood gas analysis by a dose-dependent correlation with IAP and time (figure 2). Most notably, a decrease was seen in median pH (ranging from 7.27 in the control group to 6.86 in the highest IAP group; figure 2A) and median pO_2_ (ranging from 503 mm Hg to 192 mm Hg; figure 2B) at 180 min. pH and pCO_2_ values were negatively correlated with IAP at that time point (Rs=−0.934, p<0.001 and Rs=−0.752, p<0.001 respectively). Bicarbonate levels and oxygen saturation also decreased, and demonstrated a negatively correlation with IAP (figure 2D and F). Positive correlations were seen between IAP and pCO_2_ (Rs=0.882, p<0.001; figure 2C) and base deficit (Rs=0.862, p<0.001; figure 2E) at 180 min. Median pCO_2_ increased over time with a range from 44.2 mm Hg in the control group to 99.6 mm Hg in the highest IAP group, and median base deficit increased with a range from 5.3 to 11.9 for the same groups at 180 min.

### Outcome characteristics

**Figure 3:**
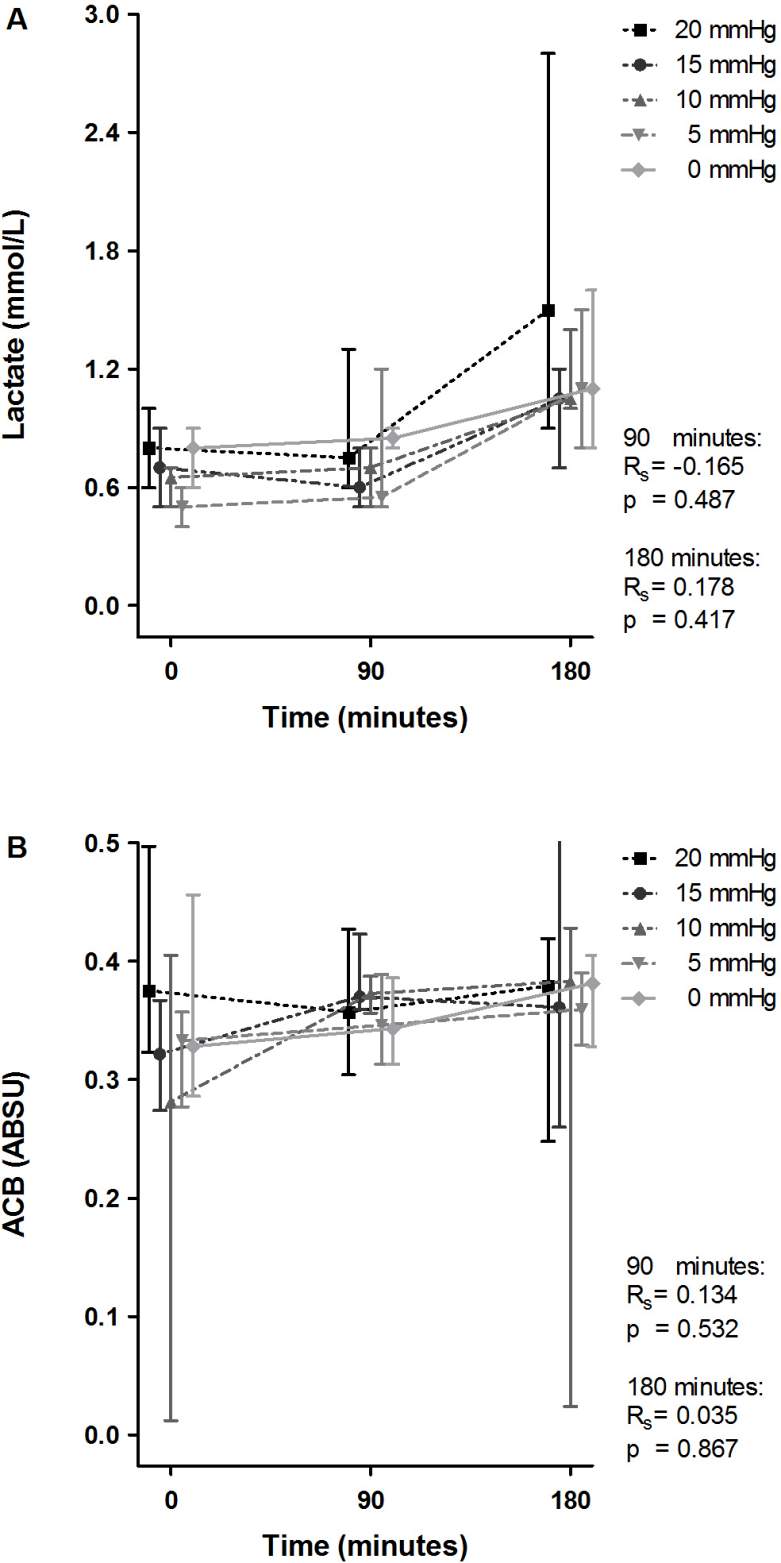
Effect of intra-arterial pressure (IAP) increase on serum lactate and albumin-cobalt binding at 90 and 180 min. ABSU, absorbance units; ACB, albumin-cobalt binding capacity. Lactate (A) and albumin-cobalt binding (B) at 0, 90, and 180 min are demonstrated for the individual groups and displayed as median with upper and lower limits. The Spearman correlation coefficient (Rs) and p value (p) represent correlation between IAP and the individual variables with corresponding statistical significance at 90 and 180 min.

**Figure 4:**
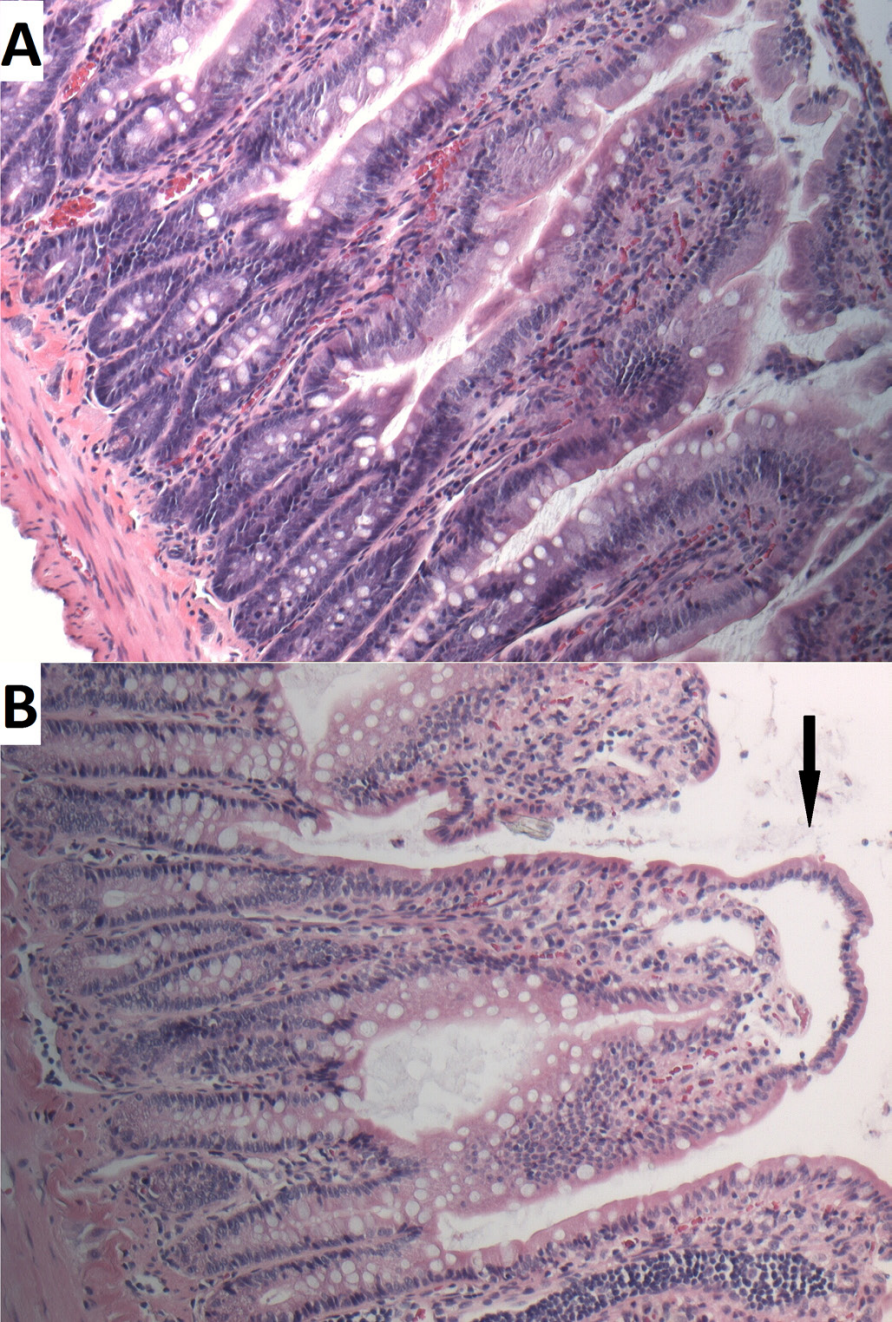
H&E sections of the least and most extensively damaged mucosa. In most sections of rats in all groups, no lesions were found in the small intestine (A). Grade 3 Parks/Chiu lesions of ischemia (ie, mucosal lifting down sides of the villi; see arrow) were seen sporadically (B).

During the last phase of this experiment, serum lactate levels increased. No significant correlation was found between this and IAP (Rs=0.178, p=0.417; figure 3A). Although a decrease was seen in ACB capacity (demonstrated by an increase of ABSU), group medians did not reach the threshold of 0.4 ABSU for systemic ischemia. No correlation between ACB capacity and IAP was observed (figure 3B). This finding was confirmed by histopathological examination, and no evident signs for ischemic damage were found in the H&E-stained sections (figure 4). The Parks score was comparable in all groups: no inflammation or necrosis was found in any of the specimens. The Park/Chiu score for mucosal lifting ranged from 0 to 4; no significant correlation between Park/Chiu score and IAP was found (Rs=−0.141, p=0.501).

## Discussion

In this model of IAH, an increase in IAP did not result in significant ischemic complications within the first 3 hours. Even though a relation was seen between increasing IAP and hemodynamic deterioration, no histopathologic signs of irreversible damage to the intestines were found; nor were there any signs of systemic ischemia throughout the experiment as tested using the ACB assay. This is in contrast with the results of the only other similar study in rabbits. In this study, rabbits were exposed to an IAP of 25 mm Hg for 1 hour. During this period, histopathology and the ACB assay demonstrated a statistically significant increase in intestinal ischemia.^13^

The findings of this study may indicate that in the earliest phase of increasing IAP, physicians have some time to focus on adequate respiratory and hemodynamic support before preventive open abdomen decompression is applied. In the first phase of IAH, respiratory deterioration may already be very profound without causing irreversible damage to the intestines. During this period, the possible effects of less-invasive measures can be awaited with no further harm being done. This supports the theory that effects of percutaneous catheter decompression (PCD) can be awaited for a period of 4 hours; if PCD was not effective, urgent open abdomen decompression should be initiated.^7^

The respiratory and hemodynamic deterioration observed in this study demonstrated the suitability of the model used. Apart from the metabolic acidosis in the 0 mm Hg group, outcomes seem to be an adequate reflection of IAH in humans.^17 18^ The metabolic acidosis in the 0 mm Hg group was unexpected, a finding possibly due to the rats being relatively hypovolemic. In order to keep rats as normovolemic as possible, rats in the 0 mm Hg group only received resuscitation fluids for compensation of blood collection. As confirmed by the negative CVPs, this minimal support seems to have been insufficient.

All animals were sacrificed at 180 min for histopathological evaluation; reperfusion following decompression was not awaited. Theoretically, during reperfusion, free radicals may cause significant oxidative damage.^19^ The oxidative damage might even be more extensive than the damage induced by IAH itself. Demonstrating this, however, was not the aim of this study.

Possible limitations of this study were the relatively small sample size and the small size of the animals used in this experiment. It is known that the abdominal wall elasticity of small animals significantly differs from the elasticity in humans.^20^ Even though a plaster cuff was placed around the abdomen of the rats, this may have influenced the results of the present study. Moreover, the pathophysiology of ACS in critically ill humans is likely different from ACS in the otherwise healthy rats used in the current model. Translation of this IAH model to the human situation should therefore be done with care.

The period of 180 min was relatively short compared with patients who are observed and conservatively managed after bowel obstruction or non-operatively managed blunt abdominal trauma. This is inherent to the selected rat model. From a pilot study, it was known that exposing the rats to IAP levels of 25 or 30 mm Hg resulted in death within 1 hour. Even at 20 mm Hg IAP, the rats’ hemodynamic and respiratory parameters progressed to lethal levels, making it impossible to keep the animals alive longer than 180 min. At that time point, arterial blood gas values reached morbid levels in the highest IAP group. In one case, the rat died instantaneously when the IAP was relieved. Nevertheless, this experiment was suitable for demonstrating a principle. The outcomes of this study require confirmation in larger study groups or larger animals.

In conclusion, during this experimental study of increased IAP, no signs of early irreversible ischemic damage were found, while profound deterioration of respiratory and hemodynamic parameters were already present. These findings may indicate that in the early phase of increasing IAP, physicians have some time to focus on adequate respiratory and hemodynamic support before preventive open abdomen decompression is applied. Non-invasive measures which prevent a further increase of IAP seem preferable.
